# Effect of a Toothpaste/Mouthwash Containing *Carica papaya* Leaf Extract on Interdental Gingival Bleeding: A Randomized Controlled Trial

**DOI:** 10.3390/ijerph15122660

**Published:** 2018-11-27

**Authors:** Ina Saliasi, Juan Carlos Llodra, Manuel Bravo, Paul Tramini, Claude Dussart, Stéphane Viennot, Florence Carrouel

**Affiliations:** 1 Laboratory “Systemic Health Care”, EA4129, University of Lyon, 69008 Lyon, France; inasaliasi@yahoo.com (I.S.); claude.dussart@univ-lyon1.fr (C.D.); stephane.viennot@univ-lyon1.fr (S.V.); florence.carrouel@univ-lyon1.fr (F.C.); 2 Department of Preventive and Community Dentistry, Faculty of Odontology, University of Granada, 18010 Granada, Spain; mbravo@ugr.es; 3 Department of Public Health, Faculty of Dental Medicine, University of Montpellier, 34090 Montpellier, France; paul.tramini@orange.fr

**Keywords:** biofilm, anti-inflammatory, *Carica papaya*, interdental bleeding, natural dentifrice, sodium lauryl sulfate free dentifrice, mouthwash, essential oils

## Abstract

Clinical research on herbal-based dentifrice +/− mouth rinse products is very limited compared with the plethora of research on conventional oral care products under normal oral hygiene conditions. The aim of this study was to determine the anti-inflammatory effects of a novel plant *Carica papaya* leaf extract (CPLE) on interdental bleeding in healthy subjects. In this randomized, single-blind parallel-design study, the eligible subjects were generally healthy non-smokers, aged 18–26, who exhibited healthy periodontal conditions upon study entry. The participants were equally randomized into the following four groups: CPLE dentifrice, CPLE dentifrice and mouthwash, sodium lauryl sulfate (SLS)-free enzyme-containing dentifrice and SLS-free enzyme-containing dentifrice with essential oil (EO) mouthwash. Subjects were instructed to brush their teeth twice a day without changing their other brushing habits. Interdental bleeding (BOIP) was measured from inclusion (T_0_) until the fourth week (T_4_) of the study. Clinical efficacy was assessed after one, two, three and four weeks of home use. The analyses compared BOIP between groups and were then restricted to participants with ≥70% and then ≥80% bleeding sites at T_0_. Pairwise comparisons between groups were performed at T_0_ and T_4_, and a logistic regression identified correlates of gingival bleeding (T_4_). Among 100 subjects (2273 interdental sites), the median percentage of bleeding sites per participant at T_0_ was 65%. The bleeding sites dramatically decreased in all groups between T_0_ and T_4_ (relative variations from −54% to −75%, *p* < 0.01 for all). Gingival bleeding did not significantly differ between the CPLE dentifrice and the SLS-free dentifrice +/− EO mouthwash groups (from *p* = 0.05 to *p* = 0.86), regardless of the baseline risk level. Among the CPLE dentifrice users, fewer bleeding sites were observed when toothpaste and mouthwash were combined compared to bleeding sites in those who used toothpaste alone (21% vs. 32%, *p* = 0.04). CPLE dentifrice/mouthwash provides an efficacious and natural alternative to SLS-free dentifrice +/−EO-containing mouthwash when used as an adjunct to mechanical oral care to reduce interdental gingival inflammation.

## 1. Introduction

The self-realized mechanical disruption of biofilm with tooth brushing and interdental tooth brushing is the best prevention method currently to prevent and reduce gingival inflammation [1]. This mechanical action is insufficient without the use of chemical products as dentifrices. Chemicals agents, such as triclosan, sodium lauryl sulfate (SLS), and propylparaben, and allergens such as methylisothiazolinone and methylchloroisothiazolinone, as well as chlorhexidine, have been added to dentifrices to reinforce their antibacterial action. These products can pose a human health risk [2,3,4,5]. Some of these substances show undesirable side effects, such as altered taste and tooth staining, and doubts persist regarding detrimental impacts on endocrine function, notably, fertility [6,7]. Some manufacturers have moved away from SLS, chlorhexidine and triclosan and introduced other, less irritating surfactants such as non-ionic polyethylene glycol ethers of stearic acid [8,9]. Likewise, enzyme-containing dentifrices, such as Zendium^®^, Enzycal^®^, and Jason Powersmile Toothpaste^®^, which are free of SLS, chlorhexidine and triclosan, have also been developed as alternatives to dentifrice chemotherapeutic agents. Antimicrobial agents present in dentifrices cannot effectively penetrate hard-to-reach areas in the oral cavity, resulting in biofilm-dwelling bacteria accumulation in the interdental spaces [10,11]. In this sense, mouthwashes for daily use are a complement to brushing to improve oral health [12,13].

It is necessary to distinguish solutions to be used daily, after or before brushing of the teeth, from those with a therapeutic aim, which often have the status of a drug and whose use must be much more punctual. These therapeutic solutions are usually prescribed following oral surgery, tooth extraction or device-related injury [14,15]. Due to the high dose of chlorhexidine (0.2% minimum), these therapeutic solutions are also are good antiseptics. The effects of an alcohol vehicle solution versus an essential oil (EO) mouthwash (for example thymol, menthol, eucalyptol and methyl salicylate) as daily mouthwashes in relation to antiplaque and antigingivitis properties have been discussed [16]. An unanswered question is whether toothpastes and mouthwashes used in combination have a significant effect on inflammation parameters, without causing any interference regarding their inhibitory effects on plaque, regardless of the order of use [17].

More consumers are using natural health products in the modern world [18]. In recent years, natural compound (excluding EO)-containing mouthwashes have shown a growth in demand in markets and the professional community [19]. A growing number of dentists have embraced the philosophy that natural agents are better for the oral health of children and the general population [20]. Given the increased bacterial resistance to antibiotics currently used in dentistry, natural compounds are important for the prevention of oral bacterial growth, adhesion and colonization [21]. Herbal medicines, including herbs, herbal materials, herbal preparations, finished herbal products that contain parts of plants or other plant materials as active ingredients, and various medicinal plants, individually or in combination, have been used for over 2000 years to maintain oral hygiene and to prevent inflammation [22,23,24]. An overview of representative plant extracts found that they have favorable antimicrobial properties against oral bacteria [25]. Side-effect-free medicinal herbs–*Acacia chundra* Willd, *Adhatoda vasica* Nees, *Mimusops elengi* L., *Piper nigrum* L., *Pongamia pinnata* (L.) Pirerre, *Quercus infectoria* Oliv, etc.—might supplement or even be a substitute for conventional anti-infectious agents in the battle against periodontitis and other biofilm-related diseases [26,27,28]. The antimicrobial properties of herbal dentifrices and mouthwashes vary greatly; however; few of these products have undergone rigorous testing, as evidenced by the limited amount of information on their safety and efficacy in the literature [29].

Dentifrices labelled as “natural” typically do not include ingredients such as synthetic sweeteners, artificial colors, preservatives, additives, or synthetic flavors and fragrances [30]. Clinical research on herbal-based mouth rinses and dentifrices is very limited, whereas a plethora of research exists on conventional oral care products [27]. Among these products, natural dentifrices/mouthwashes, in the form of water mixed with a powder containing *Carica papaya* leaf extract (CPLE) have been recently marketed in Europe (Gencix^®^). However, to the best of our knowledge, there have been no studies investigating the anti-inflammatory action of CPLE.

The main objective of this study was to compare the efficacy in reducing interdental gingival bleeding between a natural dentifrice/mouthwash containing CPLE and a classical SLS-free enzyme-containing dentifrice, alone or associated with an EO mouthwash.

## 2. Materials and Methods

### 2.1. Study Design

The study was designed as a single-blind, four-group, randomized, controlled, parallel clinical trial. The guidelines of the CONSORT Statement were followed. The study protocol was reviewed and approved by the Institutional Ethics Board Committee on research involving humans, Dental Faculty, University of Granada, Spain. Written informed consent in agreement with the Declaration of Helsinki was obtained from all enrolled individuals. Clinical Trials Registry—2018-000905-22. Registered 28 February 2018 (retrospectively registered).

The eligible volunteer participants were randomly assigned to one of the four experimental groups, each of which included 25 participants using a random number generator (www.random.org). To achieve the same sample size in both groups and simultaneously satisfy the randomization procedure to achieve balanced groups with respect to the most relevant variables (sex and baseline bleeding), a stratified (two levels for sex and two levels for baseline bleeding) block randomization (computer-assisted) method was used. Baseline bleeding was defined as the percentage of bleeding sites per participant as follows: a high level of bleeding if the participant had ≥30% bleeding sites and a low level of bleeding if the participant had <30% bleeding sites [31]. Each participant was identified using a code. The methods were not changed after the trial began.

### 2.2. Participants

The source population consisted of voluntary healthy subjects aged 18–26. The respondents were assured that participation was voluntary. One hundred and forty-one persons volunteered to participate in the study. All of the candidates were screened for suitability by the research team. The subjects who met the following inclusion criteria were included in this study. The selection inclusion criteria were (i) 18–26 years old; (ii) good general health and not pregnant or breastfeeding; (iii) the presence of at least 20 natural teeth (excluding 3rd molars); (iv) non-smokers; (v) willing to give written formed consent; (vi) capable of following the study for a period of 4 weeks; (vii) no allergy to personal care products or their ingredients; (vii) willing to abstain from the use of interdental brushing devices (interdental brushes, dental floss, etc.) during the study; (ix) no implants or orthodontic appliances; (x) willing to refrain from the use of brushing supplements containing antibacterial agents such as amine fluoride, chlorhexidine, silver ions, etc. during the study; and (xi) brushing the teeth at least twice per day.

**Figure 1:**
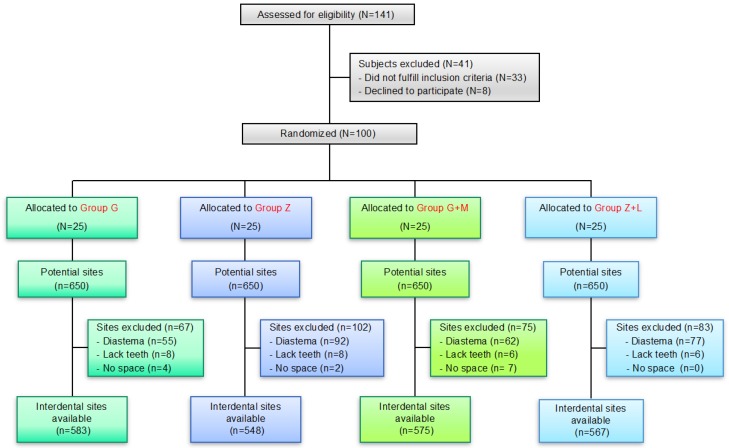
Flow chart of the study.

The exclusion criteria were (i) subjects unable or unwilling to sign the informed consent form, (ii) unable to answer questions, (iii) non-cooperative, (iv) a state of health that requires premedication before visits or dental procedures, (v) subjects with the following pathologies: diabetes, haemophilia, anticoagulant treatment, and risk for infectious endocarditis, (vi) the presence of moderate or advanced periodontal disease, (vii) 2 or more decayed teeth during the screening or other diseases of the hard or soft oral tissues, (viii) use of drugs that affect salivary flow, (ix) use of antibiotics or antimicrobial drugs within 30 days prior to the study visit, (x) participation in any other clinical study in 1 week prior to enrolment in this study, (xi) subjects requiring dental treatment or other oral prophylaxis during the study dates, (xii) allergy to dentifrice ingredients, (xiii) allergy to several mouthwash components, (xiv) the presence of an orthodontic appliance, (xv) a history of allergy to natural remedies such as herbal ingredients, (xvi) immune deficiencies, (xvii) smokers (daily consumption greater than or equal to 1 cigarette), (xviii) regular use (more than once a week) of interdental brushes or dental floss in addition to dental brushing, (xix) regular (more than once a week) use of a mouthwash and (xx) subjects requiring geographical mobility. All eligible volunteers were given oral information about the products and the purpose of the study. All subjects could at any time withdraw from the study. The study flow chart is shown in Figure 1.

### 2.3. Interventions

The study was conducted in the Division of Prevention, Dental Clinic, University of Granada, Spain. At baseline, April 2017, the prescreened participants were referred to the clinic for examinations (gingivitis, periodontal conditions, and bleeding). A colorimetric probe (IAP Curaprox; Curaden, Kriens, Switzerland) was used to evaluate the diameter of the interproximal spaces, except those between the second and third molars, to determine the appropriate size of the interdental brushes (IDBs) for each site [32]. After the baseline oral examinations and assessments of the other inclusion/exclusion criteria, the qualifying participants were randomly assigned to one of the following four groups (Figure 1): (i) Group G: test CPLE dentifrice; (ii) Group G + M: test CPLE dentifrice with CPLE mouthwash; (iii) Group Z: control with SLS-free enzyme-containing dentifrice; (iv) Group Z + L: control with SLS-free enzyme-containing dentifrice and alcohol-based EO mouthwash.

All participants were provided with sufficient amounts of their assigned products. They were asked to brush for the total duration of the study twice daily for 2 min using only their provided toothbrush and assigned dentifrice/mouthwash. Groups G + M and Group Z + L were directed to use 20 mL of CPLE or alcohol-based EO mouthwash twice a day for 30 s after tooth brushing. The first rinse and rinses on the examination visits occurred under supervision at the study site. All other rinsing was unsupervised. Subsequent rinsing with water was not allowed. The use of any other dental products or interdental cleaning aids during the study was not allowed. 

### 2.4. Description of Dentifrices and Mouthwashes

The CPLE dentifrice and mouthwash were used after dilution of the powder in water. The powder contained an aqueous extract of *Carica papaya* leaves (40%) with pumice (60%) (Gencix^®^, Esprit d’Ethique, Orvault, France).

The SLS-free enzyme-containing dentifrice contained sodium fluoride, colostrum, lactoperoxidase, lysozyme glucose oxidase and amyloglucosidase (Zendium^®^ classic, Sara Lee, Amersfoort, The Netherlands).

The alcohol-based EO mouthwash contained eucaliptol (0.092%), menthol (0.042%), methyl salicylate (0.060%), and thymol (0.064%) as active ingredients. Inactive ingredients included water, alcohol (21.6%), benzoic acid, poloxamer 407, benzoic acid, and flavoring (Listerine^®^ Cool Mint^®^, Manufacturer Johnson & Johnson Healthcare Products, Maidenhead, UK). 

### 2.5. Assessment and Outcome

All selected individuals were followed after the inclusion visit (T_0_) for 4 weeks with the following weekly dental consultations: Week 1 (T_1_), Week 2 (T_2_), Week 3 (T_3_) and Week 4 (T_4_). These consultations involved the visual assessment of interdental gingival bleeding and supragingival plaque (PI) by a trained and calibrated dental examiner. 

Gingivitis was assessed using the Bleeding on Interdental Brushing Index (BOIP) on the interdental space. All interdental sites were recorded, as was the bleeding response to the horizontal pressure applied in the interdental area by a calibrated IDB. After 30 s, bleeding at each gingival unit was recorded according to the following scale: 0, absence of bleeding; and 1, bleeding. The probing protocol was always the same, starting in the 16–17 interdental space and finishing in the 46–47 interdental space.

At each visit during the evaluation period, a calibrated interdental brush was introduced into the interproximal space, and the presence of bleeding was observed. The pressure applied by a horizontal brush in the interdental area should be firm and continuous until reaching maximum compression with minimal discomfort to the patient. The pressure used to place the IDB was approximately 50–100 N·cm^−2^ (0.20–0.40 gram-force), and 83% of participants were assigned a Visual Analogue Scale score of ≤1. More details can be found in a previous study [31].

The plaque score was assessed using a standard scale called the Turesky Modification of the Quigley Hein Plaque Index after disclosing and scored from 0 to 5, where 0 = no plaque, 1 = separate flecks or a discontinuous band of plaque at the gingival (cervical) margin, 2 = a thin (up to 1 mm), continuous band of plaque at the gingival margin, 3 = a band of plaque wider than 1 mm but less than 1/3 of the surface, 4 = plaque covering 1/3 or more, but less than 2/3, of the surface, and 5 = plaque covering 2/3 of more of the surface [33].

The primary efficacy endpoint was the percentage of bleeding sites at four weeks, and the secondary endpoints included the mean BOIP at one, two and three weeks and the mean PI at one, two, three, and four weeks.

### 2.6. Sample Size Calculation

The calculation of the sample size (interdental sites) was performed using Sample Power 2.0 (SPSS, Chicago, IL, USA) software. A *t*-test for independent groups was used to detect a power of 80% and 5% alpha error for an estimated Cohen’s d of 0.5, a medium effect size according to Cohen’s scale, for bleeding after interdental brush use.

We required 256 a-priori interdental sites (64 per group, 4 groups). After considering a design effect (owing to interdental sites being clustered within participants) of 5.0 (estimated from previous experience with these IDBs) in estimating the bleeding percentage, the sample size increased to 1280 interdental sites (=256 × 5). Furthermore, after considering that an estimated 80% of the interdental sites would be available for analysis (i.e., 20% would be excluded owing to lack of space to introduce the IDB, the presence of diastema or missing teeth), the sample size increased to 1600 interdental sites (=1280/0.80). Considering 26 a-priori interdental sites per participant, this resulted in a minimum sample size of 61 participants (i.e., approximately 15 participants per group). 

### 2.7. Data Analysis 

The output variable is the level of interproximal bleeding at the interproximal site after one of four commercially available dentifrices/mouthwashes. The statistical unit was the individual interdental site with the available BOIP measurement. After the preliminary descriptive analyses, the proportion of the individual bleeding interdental sites among the treatment groups was compared. Standard errors (se) were calculated according to Dubey and colleagues’ method [34]. In parallel, relative changes in the proportion of bleeding sites from T_0_ to T_4_ were assessed within each group. All *p*-values were corrected for complex sampling (multiple interdental sites within the mouth). Then, analyses were further conducted after restricting the data to subjects with ≥70% of bleeding interdental sites (median value). In this analysis, we used pairwise univariate comparisons, conducted weekly from baseline (T_0_) until the 4th week (T_4_). The statistical methods are indicated in the table footnotes. Given the possible loss of comparability of the randomized groups due to the restriction, a multivariate multilevel logistic model was computed at T_4_, investigating the risk of interdental bleeding as a dependent variable with treatment group (G vs. Z) as the main explanatory variable. Descriptive statistics (*p*-values and standard errors) were performed with SPSS Windows 20.0 (IBM Inc., Chicago, IL, USA) for analyses based on the patients. The SUDAAN 7.0 (RTI, RTP, NC) was used to account for clustering (multiple interproximal spaces within the patient) for analyses based on interproximal spaces. The statistical methods are indicated in the table footnotes. 

## 3. Results

### 3.1. Baseline Characteristics

#### 3.1.1. Subject Characteristics

Of the 141 subjects that volunteered, 33 were excluded based on the eligibility criteria. Eight participants dropped out for reasons unrelated to the study protocol (Figure 1). In total, 100 participants (47 males, 53 females) completed all visits of this clinical trial and had a mean age of 23.2 (±3.0) years (range: 19–33 years). All clinical parameters were normally distributed. The mean proportion of interdental bleeding sites per subject at baseline ranged from 62 to 69% in the different groups. The median proportion of bleeding sites per subject was close to 65% (Table 1). At the sampling site level, the overall plaque indexes for groups G, Z, G + M and Z + L were 0.82 ± 0.19; 0.90 ± 0.21; 0.87 ± 0.19 and 0.76 ± 0.30, respectively. There was no significant difference in plaque accumulation among the four groups or the four timepoints. The mean clinical attachment loss (CAL) values (mm) were 1.45 ± 0.20; 1.37 ± 0.24; 1.60 ± 0.32 and 1.54 ± 0.38 (*p* > 0.05), respectively. No safety issue was noticed for any patient throughout the study period.

#### 3.1.2. Interdental Sites

Of the 2600 a-priori interdental sites available (i.e., 100 subjects × 26 interproximal sites/subjects), 13 were excluded due to lack of space to introduce an IDB, 28 for missing teeth and 286 for diastema, leaving a total of 2273 interdental sites available for the analyses (Figure 1). The distribution of interdental sites according to location was upper-posterior (n = 752, 33.1%), lower-posterior (n = 757, 33.3%), upper-anterior (n = 392, 17.2%) and lower-anterior (n = 372, 16.4%). According to the IDB diameter, the distributions were 1.1 mm (n = 88, 3.9%), 0.9 mm (n = 284, 12.5%), 0.8 mm (n = 589, 25.9%), 0.7 mm (n = 720, 31.7%) and 0.6 mm (n = 592, 26.0%). There were no significant differences among the groups for the site location (*p* = 0.30) or the IDB diameter (*p* = 0.35, *p*-values calculated with chi-square test, corrected for multiple sites within the patients with CROSSTAB in SUDAAN 7.0).

### 3.2. Inferential Analyses

#### 3.2.1. Changes in Gingival Bleeding in Each Group from T_0_ to T_4_

**Figure 2:**
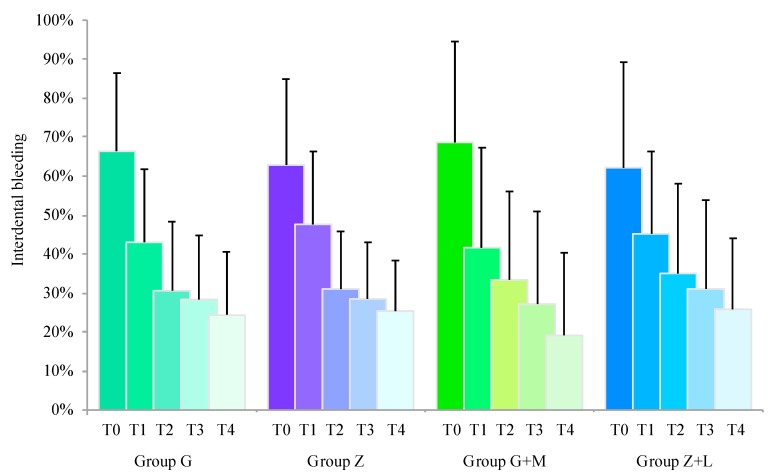
Evolution of interproximal bleeding by group in 2273 interdental sites (100 subjects). Group G: test *Carica papaya* leaf extract (CPLE) dentifrice; Group G + M: test CPLE dentifrice with CPLE mouthwash; Group Z: control with sodium lauryl sulfate (SLS)-free enzyme-containing dentifrice; Group Z + L: control with SLS-free enzyme-containing dentifrice and alcohol-based essential oil mouthwash; T_0_: basal; T_1_: 1 week; T_2_: 2 weeks; T_3_: 3 weeks; T_4_: 4 weeks.

A salient monotonous decrease in bleeding of interdental sites was observed between T_0_ and T_4_ in subjects from all groups, regardless of the baseline interdental bleeding level (Figure 2). Overall, the proportion of bleeding sites per subject substantially decreased in all groups from −59% to −72%, (*p* < 0.01 for all), although this trend was more pronounced in Group G + M (Table 2).

After restricting the analysis to patients with ≥70% bleeding sites at T_0_, the magnitude of the decrease from T_0_ to T_4_ was slightly higher in all groups (from −54% to −65%, *p* < 0.01 for all, Table 3). Interestingly, the proportional decrease in bleeding interdental sites over the four-week period tended to be slightly greater in subjects with 70% or more bleeding sites at baseline compared with that of the overall study population.

#### 3.2.2. Risk of Interdental Bleeding between Treatment Groups

Overall, we did not observe any significant difference between Group G and Group Z or Group Z+L in terms of a reduction in gingival bleeding over the four-week period (Table 2). In the subgroup presenting with ≥70% bleeding sites, a near-significantly lower proportion of bleeding interdental sites was observed in subjects using CPLE dentifrice combined with mouthwash compared to those receiving SLS-free enzyme-containing dentifrice with alcohol-based EO mouthwash (21% vs. 33%, *p* = 0.05, Table 3). More importantly, this trend disappeared when the combined use of CPLE dentifrice was compared to the use of SLS-free enzyme-containing dentifrice alone. Furthermore, a near-significantly lower interdental bleeding level was seen when using CPLE dentifrice and mouthwash compared to the combined use of SLS-free enzyme-containing dentifrice and alcohol-based EO mouthwash. In contrast, no marked difference was observed in between-group pairwise comparisons. A unique and statistically significant between-group difference was observed at 4 weeks in the high-risk category between subjects receiving CPLE dentifrice with or without mouthwash. In parallel, the decrease in bleeding interdental sites from T_0_ to T_4_ was more prominent in Group G+M than that in Group G (−75% vs. −62%, *p* = 0.04).

No significant difference was observed between the different high-risk groups for the studied variables and interproximal bleeding at T_4_ (Table 4).

## 4. Discussion

This study compared the effectiveness of a natural dentifrice containing *Carica papaya* leaf extract to a commercially available SLS-free enzyme-containing dentifrice in reducing interdental gingival bleeding. Since mouthwashes are typically used in conjunction with a mechanical cleaning regimen, the effects of CPLE dentifrice used alone or with CPLE mouthwash versus an antimicrobial EO mouthwash in combination with a non-antimicrobial dentifrice were also assessed.

To the best of our knowledge, this is the first clinical trial on the effects of the application of natural extracts versus SLS-free enzyme-containing dentifrices against the interdental inflammatory process. Our study reveals that CPLE dentifrice with or without mouthwash and SLS-free enzyme-containing dentifrice with or without alcohol-based EO mouthwash significantly reduced interdental inflammation at four weeks. Moreover, no significant difference was observed between the CPLE dentifrice and the SLS-free enzyme-containing dentifrice whether or not they were combined with the alcohol-based EO mouthwash. A dramatic decrease in interdental gingival bleeding appeared within the four-week period in all groups. In the subjects with more than 70% bleeding, the combined use of the CPLE dentifrice, both as a dentifrice and a mouthwash, showed a greater impact on bleeding reduction than the use of the CPLE dentifrice alone.

Additionally, the two dentifrices tested significantly decreased interdental inflammation but through different actions. Dentifrice containing enzymes stimulated natural salivary defenses. The enzyme cascade leads to the production of hypothiocyanite and hydrogen peroxide, which could result in an increase in oxygen levels and an inhibition of the growth of anaerobic bacteria. This result promotes a shift in the bacterial community, resulting in an increase in bacteria associated with gum health and a concomitant decrease in those associated with periodontal disease, such as Treponema spp [35]. No salient impact on interdental bleeding of the addition of mouthwash with the dentifrice was observed in low or high-risk subjects. Although no formal conclusion was possible given the non-significant differences, the results tended to be better with the use of the SLS-free enzyme-containing dentifrice alone. Components of the alcohol solution may interact [36]. In the present study design, it can be concluded that the interdental bleeding efficacy of a post-brushing alcohol-based EO or CPLE mouthwash does not seem to be reduced under the influence of a normal tooth brushing regimen with a dentifrice, whether or not the dentifrice contained enzymes or CPLE powder.

The mechanisms of action of the CPLE dentifrice in the process of reducing interdental bleeding have not yet been clearly identified. The various organs of this plant are rich in specific compounds. For example, papain, an enzyme from papaya, is mainly harvested from the fruit latex, because it does not accumulate to sufficient levels in other tissues [37]. Additionally, extraction solvents can be concentrated in certain compounds to a greater or lesser extent. Polar solvents, such as water, concentrate the most polar molecules, whereas highly apolar solvents, such as dichloromethane, concentrate apolar molecules [38]. Enzyme effects are unlikely because the aqueous extraction by decoction includes heating, which would unfold this type of molecule. Vascular effects have not been documented in the *papaya* leaf to our knowledge. Previous studies have discovered several activities from the leaves of *Carica papaya* linked to a few identified metabolites such as amino acids, fatty acids, sugars and organic acids [39,40]. Therefore, the observed activities are probably related to terpenes, which have been found to have antibacterial activity [41] and antifungal activity [42]. Another possibility is the large family of phenolic compounds that can have antioxidant activity [43,44,45,46], antitumour activity [43,47] or immunomodulatory and antithrombocytopenic activity [48]. Similarly, the biological effects of flavonoid secondary metabolites found in papaya leaves are evident. The anti-inflammatory action (anti-TNF-alpha) of CPLE dentifrice could explain the global reduction of gingival inflammation and thus the reduction of interdental bleeding. Anti-inflammatory activity described by Erlund and colleagues is related to quercetin, hesperitin and naringenin present in the leaves [49]. The anti-inflammatory mechanism of quercetin induces the activation of extracellular signal kinase (ERK), c-Jun NH2-terminal kinase (JNK), c-Jun and nuclear factor-κB (NF-κB), or it induces an increase in peroxisome proliferator-activated receptor (PPARγ) activity [50]. In addition, antimicrobial activity has also been reported, including 3 *Candida* species and Gram-positive and Gram-negative bacteria [51,52,53]. The final family of metabolites identified are the alkaloids, which have cytotoxic activities [47,48,54,55], especially with certain cell lines. The literature also reveals antimicrobial activities [37], particularly antifungal [42,56,57], antiparasitic [58,59,60,61], antibacterial [41,62,63,64,65] and antiviral activities [66,67]. Finally, anti-inflammatory action has been attributed to the alkaloids of papaya [68,69].

Some limitations of this study must be acknowledged. The stratification of the analyses of the baseline interdental bleeding may have affected the comparability initially obtained between groups after randomization. Nonetheless, groups did not statistically differ at the baseline compared to the outcome, particularly among subjects presenting with a higher baseline interdental bleeding risk.

The subjects’ actual adherence to the assigned therapies was unknown. Nonetheless, a dramatically significant decrease in bleeding at interdental sites was observed in all groups, suggesting a global correct adherence level. This was a single-blind study, which could have impacted some of the comparisons between groups. Nonetheless, we believe that our findings were minimally affected by such bias, as our outcome was collected by trained investigators who were unaware of the subjects’ exposure. Further investigations, particularly observational studies, will be eventually needed to confirm our conclusions in real-life settings.

While the antimicrobial effect of only Listerine Cool Mint with a vehicle alcohol solution was tested in combination with SLS-free enzyme-containing dentifrice, the results of our study may not be extrapolated to all Listerine products [70]. For instance, alcohol-based EO mouth rinse penetrates and kills microorganisms deeper and more effectively in plaque biofilm in typical exposure times when compared to dentifrice chemotherapeutic agents [10].

Our results have clinical implications. CPLE dentifrice and mouthwash provide an efficacious alternative to SLS-free enzyme-containing dentifrice with or without EO-containing mouthwash when used as an adjunct to mechanical oral care. The data suggest that the CPLE dentifrice has similar benefits and efficacy as the SLS-free enzyme-containing dentifrice alone or associated with an alcohol-based EO mouthwash regarding interdental bleeding. Furthermore, our findings indicate that the optimal benefit of the CPLE dentifrice in reducing interdental bleeding occurs when it is used as both a dentifrice and a mouthwash. Given the current concerns regarding the safety of many commercial types of dentifrice, the CPLE dentifrice could constitute an interesting natural alternative to treat susceptible persons with interdental bleeding, particularly at an advanced stage of gingival bleeding (>70%).

Nonetheless, our findings require confirmation in a less selected population or in patients with periodontitis. Further comparisons against classical dentifrices and with other periodontitis outcomes are also desirable. Our findings indicate that CPLE dentifrice/mouthwash used alone is safe. However, a natural product does not inherently mean the absence of any adverse outcomes. The benefit risk balance of CPLE dentifrice use should be investigated more thoroughly. Likewise, the cost effectiveness ratio of CPLE dentifrice use must not be overlooked. Further research should be done, especially on indications for long-term use and maintenance of a healthy oral microbiota as well as special needs patients.

## 5. Conclusions

Finally, we conclude that the *Carica papaya* leaf extract dentifrice is effective when compared to an SLS-free enzyme-containing dentifrice in the reduction of gingival bleeding and inflammation and could constitute a valid parallel alternative to classical commercial dentifrices. This effect was sustained with a continuing trend of reduction observed for tissue bleeding metrics. The addition of alcohol-based EO mouthwash to the SLS-free enzyme-containing dentifrice did not show any clinical benefit in terms of the reduction in interdental bleeding.

## Figures and Tables

**Table 1:** Distribution of subject characteristics at baseline (n = 100).

Variable	Group G (N = 25)	Group Z (N = 25)	Group G + M (N = 25)	Group Z + L (N = 25)	Global *p*-Value
Sex, N (%)					0.932 ^a^
Male	11 (44.0)	13 (52.0)	12 (48.0)	11 (44.0)	
Female	14 (56.0)	12 (48.0)	13 (52.0)	14 (56.0)	
% Bleeding sites, mean ± sd	66 ± 20	63 ± 26	69 ± 22	62 ± 27	0.759 ^b^
Subject Bleeding, N (%)					
≥30% Bleeding sites	23 (92.0)	22 (88.0)	24 (96.0)	22 (88.0)	0.719 ^a^
≥70% Bleeding sites	12 (48.0)	11 (44.0)	15 (60.0)	12 (48.0)	0.700 ^a^

^a^ Chi-square test. ^b^ Analysis of variance.

**Table 2:** Interproximal bleeding after IDB (interdental brush) according to treatment group in 2273 interproximal sites ^a^ (from 100 patients).

Time	Group G			Group Z			Group G + M			Group Z + L			Paired Comparisons (*p*-Value) ^c^					
	n	% ± se ^b^	↓ ^c^	n	% ± se ^b^	↓ ^c^	n	% ± se ^b^	↓ ^c^	n	% ± se ^b^	↓ ^c^	Z-G	GM-G	ZL-G	Z-GM	ZL-GM	ZL-Z
T_0_ (Baseline)	583	67 ± 4	x	548	62 ± 5	w	575	69 ± 4	w	567	63 ± 5	v	0.45	0.66	0.61	0.26	0.39	0.84
T_1_ (1 week)		44 ± 4	y		43 ± 5	x		42 ± 4	x		47 ± 4	w	0.91	0.80	0.56	0.92	0.43	0.53
T_2_ (2 weeks)		30 ± 4	z		31 ± 4	y		35 ± 3	y		37 ± 4	x	0.85	0.34	0.21	0.52	0.62	0.33
T_3_ (3 weeks)		29 ± 3	z		29 ± 4	y		29 ± 3	y		33 ± 5	xy	1.00	0.99	0.43	0.99	0.43	0.48
T_4_ (4 weeks)		25 ± 3	z		24 ± 4	z		19 ± 2	z		26 ± 4	z	0.85	0.15	0.83	0.31	0.13	0.71
Effect. T_4_, % (95%-CI) ^e^	63 (53–72)			61 (47–75)			72 (63–82)			59 (45–73)								

^a^ 100 patients × 26 interproximal sites/patient = 2600 interproximal sites. We excluded 13 for lack of space to introduce an IDB -interdental brush-, 30 for missing teeth and 284 for diastema, leaving 2273 for the analysis (i.e., with enough interproximal space to introduce the IDB). ^b^ Standard errors (se) corrected for complex sampling (multiple interproximal spaces within the mouth) with DESCRIPT procedure in SUDAAN 7.0. ^c^ Paired comparisons were calculated correcting for complex sampling, i.e., with DESCRIPT in SUDAAN. Different letters indicate significantly (*p* < 0.05) different groups. ^d^ *p*-values corrected for complex sampling (multiple interproximal spaces within the mouth), by using DESCRIPT procedure in SUDAAN 7.0. ^e^ Percent difference between T_4_ and T_0_ = [((T_0_ − T_4_)/T_0_) × 100], with 95%-CI calculated according to Dubey et al. [2].

**Table 3:** Interproximal bleeding after IDB (interdental brush) according to patient’s baseline gingival bleeding and treatment group in 2273 interproximal spaces (from 100 patients).

Patient’s Bleeding Time	Group G			Group Z			Group G + M			Group Z + L			Paired Comparisons (*p*-Value) ^c^					
	n	% ± se ^a^	↓ ^b^	n	% ± se	↓ ^c^	n	% ± se	↓ ^c^	n	% ± se	↓	Z-G	GM-G	ZL-G	Z-GM	ZL-GM	ZL-Z
**Patient’s Bleeding <70% (50 Patients, with 1119 Spaces)**																		
T_0_ (Baseline)	297	51 ± 4	x	322	46 ± 5	x	216	46 ± 5	x	284	40 ± 5	x	0.43	0.47	0.09	0.98	0.42	0.42
T_1_ (1 week)		44 ± 4	x		39 ± 5	x		32 ± 6	xy		36 ± 5	x	0.78	0.25	0.45	0.36	0.66	0.62
T_2_ (2 weeks)		30 ± 4	y		27 ± 5	y		30 ± 5	y		25 ± 4	y	0.94	0.75	0.74	0.68	0.50	0.79
T_3_ (3 weeks)		29 ± 3	y		22 ± 5	y		20 ± 3	z		21 ± 4	y	0.56	0.36	0.44	0.80	0.94	0.87
T_4_ (4 weeks)		25 ± 3	y		20 ± 4	y		16 ± 5	z		19 ± 4	y	0.70	0.77	0.86	0.56	0.68	0.83
Effect. T_4_, % (95%-CI) ^d^	65 (52–78)			56 (36–77)			65 (43–86)			54 (33–74)								
**Patient’s Bleeding ≥70% (50 Patients, with 1145 Spaces)**																		
T_0_ (Baseline)	286	84 ± 2	x	226	85 ± 4	w	359	84 ± 2	w	283	87 ± 3	w	0.74	≈1	0.38	0.75	0.40	0.72
T_1_ (1 week)		46 ± 5	y		48 ± 9	x		48 ± 4	x		58 ± 5	x	0.84	0.72	0.09	0.97	0.14	0.32
T_2_ (2 weeks)		34 ± 5	z		38 ± 8	y		38 ± 3	y		50 ± 6	y	0.62	0.44	0.03	0.98	0.07	0.22
T_3_ (3 weeks)		32 ± 4	z		39 ± 7	y		34 ± 4	y		46 ± 7	y	0.42	0.76	0.08	0.56	0.13	0.48
T_4_ (4 weeks)		32 ± 5	z		30 ± 7	z		21 ± 3	z		33 ± 6	z	0.77	0.04	0.89	0.25	0.05	0.70
Effect T_4_, % (95%-CI)	62 (51–72)			65 (48–82)			75 (68–82)			62 (48–75)								

^a^ Standard errors (se) corrected for complex sampling (multiple interproximal spaces within the mouth) with DESCRIPT procedure in SUDAAN 7.0. ^b^ Paired comparisons were calculated correcting for complex sampling, i.e., with DESCRIPT in SUDAAN. Different letters indicate significantly (*p* < 0.05) different groups. ^c^ *p*-values corrected for complex sampling (multiple interproximal spaces within the mouth), by using DESCRIPT procedure in SUDAAN 7.0. ^d^ Percent difference between T_4_ and T_0_ = [((T_0_ − T_4_)/T_0_) × 100], with 95%-CI calculated according to Dubey et al. [2].

**Table 4:** Distribution of variables at interdental site level and multivariate associations in the different high-risk groups between the studied variables and interproximal bleeding at T_4_ (4 weeks) (n = 2273 interdentals sites from 100 subjects).

Variables	Subjects with ≥70% of Bleeding Sites at T_4_ 50 Subjects (1154 Interdental Sites)			Subjects with ≥80% of Bleeding Sites at T_4_ 31 Subjects (2273 Interdental Sites)			Subjects with ≥90% of Bleeding Sites at T_4_ 15 Subjects (344 Interdental Sites)		
	n	OR ^a^ (95%-CI ^b^)	*p*-Value	n	OR ^a^ (95%-CI ^b^)	*p*-Value	n	OR ^a^ (95%-CI ^b^)	*p*-Value
Group G	297	1.0		199			47		
Group Z	241	0.60 (0.18–1.78)	0.19	111	0.74 (0.28–2.23)	0.57	99	0.66 (0.13–3.36)	0.62
Group G + M	371	0.52 0.16–1.52)	0.07	200	0.49 (0.06–3.88)	0.16	100	0.30 (0.05–1.81)	0.19
Group Z + L	304	1.23 (0.42–3.21)	0.56	247	1.47 (0.48–4.16)	0.39	112	1.81 (0.37–8.90)	0.46

^a^ Odds ratio. ^b^ Confidence interval.

## References

[1] Van der Weijden F.A., Slot D.E. (2015). Efficacy of homecare regimens for mechanical plaque removal in managing gingivitis a meta review. J. Clin. Periodontol..

[2] Bernard A., Dornic N., Roudot A., Ficheux A. (2018). Probabilistic exposure assessment to face and oral care cosmetic products by the French population. Food Chem. Toxicol..

[3] Bourgeois D., Weiler D., Carrouel F. (2017). Oral Microbiota, Intestinal Microbiota and Inflammatory Bowel Diseases. Res. Rev. Biosci..

[4] Rompelberg C., Heringa M.B., van Donkersgoed G., Drijvers J., Roos A., Westenbrink S., Peters R., van Bemmel G., Brand W., Oomen A.G. (2016). Oral intake of added titanium dioxide and its nanofraction from food products, food supplements and toothpaste by the Dutch population. Nanotoxicology.

[5] Forte M., Mita L., Cobellis L., Merafina V., Specchio R., Rossi S., Mita D.G., Mosca L., Castaldi M.A., De Falco M. (2016). Triclosan and bisphenol a affect decidualization of human endometrial stromal cells. Mol. Cell. Endocrinol..

[6] Heringa M.B., Geraets L., van Eijkeren J.C., Vandebriel R.J., de Jong W.H., Oomen A.G. (2016). Risk assessment of titanium dioxide nanoparticles via oral exposure, including toxicokinetic considerations. Nanotoxicology.

[7] Karpuzoglu E., Holladay S.D., Gogal R.M. (2013). Parabens: Potential impact of low-affinity estrogen receptor binding chemicals on human health. J. Toxicol. Environ. Health B Crit. Rev..

[8] Sälzer S., Rosema N.A., Martin E.C., Slot D.E., Timmer C.J., Dörfer C.E., van der Weijden G.A. (2016). The effectiveness of dentifrices without and with sodium lauryl sulfate on plaque, gingivitis and gingival abrasion-a randomized clinical trial. Clin. Oral Investig..

[9] Searls J.C., Berg C.A. (1986). The influence of dentifrice detergents on oral epithelial slough. Dent. Hyg. (Chic).

[10] Serbiak B., Fourre T., Geonnotti A.R., Gambogi R.J. (2018). In vitro efficacy of essential oil mouthrinse versus dentifrices. J. Dent..

[11] Zijnge V., van Leeuwen M.B., Degener J.E., Abbas F., Thurnheer T., Gmür R., Harmsen H.J. (2010). Oral biofilm architecture on natural teeth. PLoS ONE.

[12] Haas A.N., Wagner T.P., Muniz F.W.M.G., Fiorini T., Cavagni J., Celeste R.K. (2016). Essential oils-containing mouthwashes for gingivitis and plaque: Meta-analysis and meta-regression. J. Dent..

[13] Araujo M.W.B., Charles C.A., Weinstein R.B., McGuire J.A., Parikh-Das A.M., Du Q., Zhang J., Berlin J.A., Gunsolley J.C. (2015). Meta-analysis of the effect of an essential oil-containing mouthrinse on gingivitis and plaque. J. Am. Dent. Assoc..

[14] Da Costa L.F.N.P., Amaral C.D.S.F., Barbirato D.D.S., Leão A.T.T., Fogacci M.F. (2017). Chlorhexidine mouthwash as an adjunct to mechanical therapy in chronic periodontitis: A meta-analysis. J. Am. Dent. Assoc..

[15] Barbosa M., Prada-López I., Álvarez M., Amaral B., de los Angeles C.D., Tomás I. (2015). Post-tooth extraction bacteremia: A randomized clinical trial on the efficacy of chlorhexidine prophylaxis. PLoS ONE.

[16] Lynch M.C., Cortelli S.C., McGuire J.A., Zhang J., Ricci-Nittel D., Mordas C.J., Aquino D.R., Cortelli J.R. (2018). The effects of essential oil mouthrinses with or without alcohol on plaque and gingivitis: A randomized controlled clinical study. BMC Oral Health.

[17] Elkerbout T.A., Slot D.E., Bakker E.W., Van der Weijden G.A. (2016). Chlorhexidine mouthwash and sodium lauryl sulphate dentifrice: Do they mix effectively or interfere?. Int. J. Dent. Hyg..

[18] Enioutina E.Y., Salis E.R., Job K.M., Gubarev M.I., Krepkova L.V., Sherwin C.M. (2017). Herbal Medicines: Challenges in the modern world. Part 5. Status and current directions of complementary and alternative herbal medicine worldwide. Expert Rev. Clin. Pharmacol..

[19] Tartaglia G.M., Kumar S., Fornari C.D., Corti E., Connelly S.T. (2017). Mouthwashes in the 21st century: A narrative review about active molecules and effectiveness on the periodontal outcomes. Expert Opin. Drug Deliv..

[20] Mishra R., Tandon S., Rathore M., Banerjee M. (2016). Antimicrobial Efficacy of Probiotic and Herbal Oral Rinses against Candida albicans in Children: A Randomized Clinical Trial. Int. J. Clin. Pediatr. Dent..

[21] Kouidhi B., Al Qurashi Y.M., Chaieb K. (2015). Drug resistance of bacterial dental biofilm and the potential use of natural compounds as alternative for prevention and treatment. Microb. Pathog..

[22] Varghese J., Tumkur V.K., Ballal V., Bhat G.S. (2013). Antimicrobial effect of Anacardium occidentale leaf extract against pathogens causing periodontal disease. Adv. Biosci. Biotechnol..

[23] Palombo E.A. (2011). Traditional Medicinal Plant Extracts and Natural Products with Activity against Oral Bacteria: Potential Application in the Prevention and Treatment of Oral Diseases. Evid. Based Complement. Altern. Med..

[24] Singh A., Purohit B. (2011). Tooth brushing; oil pulling and tissue regeneration: A review of holistic approaches to oral health. J. Ayurveda Integr. Med..

[25] Karygianni L., Al-Ahmad A., Argyropoulou A., Hellwig E., Anderson A.C., Skaltsounis A.L. (2016). Natural Antimicrobials and Oral Microorganisms: A Systematic Review on Herbal Interventions for the Eradication of Multispecies Oral Biofilms. Front. Microbiol..

[26] Chandra Shekar B.R., Nagarajappa R., Suma S., Thakur R. (2015). Herbal extracts in oral health care—A review of the current scenario and its future needs. Pharmacogn. Rev..

[27] Bhat S.S., Hegde S.K., Ratheesh M.S. (2014). Comparison of Antimicrobial Potential of Various Herbal Dentifrices. Int. J. Dent. Med. Res..

[28] Jayashankar S., Panagoda G.J., Amaratunga E.A., Perera K., Rajapakse P.S. (2011). A randomised double-blind placebo-controlled study on the effects of a herbal toothpaste on gingival bleeding, oral hygiene and microbial variables. Ceylon Med. J..

[29] Lee S.S., Zhang W., Li Y. (2004). The antimicrobial potential of 14 natural herbal dentifrices: Results of an in vitro diffusion method study. J. Am. Dent. Assoc..

[30] Smith C. (1996). Pasting the competition. AGD Impact.

[31] Carrouel F., Llodra J.C., Viennot S., Santamaria J., Bravo M., Bourgeois D. (2016). Access to Interdental Brushing in Periodontal Healthy Young Adults: A Cross-Sectional Study. PLoS ONE.

[32] Bourgeois D., Carrouel F., Llodra J.C., Bravo M., Viennot S. (2015). A Colorimetric Interdental Probe as a Standard Method to Evaluate Interdental Efficiency of Interdental Brush. Open Dent. J..

[33] Cugini M., Thompson M., Warren P.R. (2006). Correlations between two plaque indices in assessment of toothbrush effectiveness. J. Contemp. Dent. Pract..

[34] Dubey S.D., Lehnhoff R.W., Radike A.W. (1965). A statistical confidence interval for true per cent reduction in caries-incidence studies. J. Dent. Res..

[35] Adams S.E., Arnold D., Murphy B., Carroll P., Green A.K., Smith A.M., Marsh P.D., Chen T., Marriott R.E., Brading M.G. (2017). A randomised clinical study to determine the effect of a toothpaste containing enzymes and proteins on plaque oral microbiome ecology. Sci. Rep..

[36] Bassolé I.H., Juliani H.R. (2012). Essential oils in combination and their antimicrobial properties. Molecules.

[37] Baskaran C., Bai V.R., Velu S., Kumaran K. (2012). The efficacy of *Carica papaya* leaf extract on some bacterial and a fungal strain by well diffusion method. Asian Pac. J. Trop. Dis..

[38] Zunjar V., Mammen D., Trivedi B., Daniel M. (2011). Pharmacognostic, physicochemical and phytochemical studies on *Carica papaya* Linn. leaves. Pharmacogn. J..

[39] Gogna N., Hamid N., Dorai K. (2015). Metabolomic profiling of the phytomedicinal constituents of *Carica papaya* L. leaves and seeds by 1H NMR spectroscopy and multivariate statistical analysis. J. Pharm. Biomed. Anal..

[40] Akhila S., Vijayalakshmi N.G. (2015). Phytochemical studies on *Carica papaya* leaf juice. Int. J. Pharm. Sci. Res..

[41] Igwe O. (2015). Chemical constituents of the leaf essential oil of *Carica papaya* from sout east Nigeria and its antmicrobial activity. IJRPC.

[42] Chávez-Quintal P., González-Flores T., Rodríguez-Buenfil I., Gallegos-Tintoré S. (2011). Antifungal activity in ethanolic extracts of *Carica papaya* L. cv. maradol leaves and seeds. Indian J. Microbiol..

[43] Zuhrotun N.F., Astuti M., Murdiati A., Mubarika H.S. (2017). Anti-proliferation and Apoptosis Induction of Aqueous Leaf Extract of *Carica papaya* L. on Human Breast Cancer Cells MCF-7. Pakistan J. Biol. Sci..

[44] Inam A., Shahzad M., Shabbir A., Shahid H., Shahid K., Javeed A. (2017). *Carica papaya* ameliorates allergic asthma via down regulation of IL-4, IL-5, eotaxin, TNF-α, NF-ĸB, and iNOS levels. Phytomedicine.

[45] Zhang K., Zuo Y. (2004). GC-MS determination of flavonoids and phenolic and benzoic acids in human plasma after consumption of cranberry juice. J. Agric. Food Chem..

[46] Chen H., Zuo Y., Deng Y. (2001). Separation and determination of flavonoids and other phenolic compounds in cranberry juice by high-performance liquid chromatography. J. Chromatogr. A.

[47] Nguyen T.T.T., Shaw P.N., Parat M.O., Hewavitharana A.K. (2013). Anticancer activity of *Carica papaya*: A review. Mol. Nutr. Food Res..

[48] Fauziya S., Krishnamurthy R. (2013). Papaya (*Carica papaya*): Source material for anticancer. CIBTech J. Pharm. Sci..

[49] Anjum V., Arora P., Ansari S.H., Najmi A.K., Ahmad S. (2017). Antithrombocytopenic and immunomodulatory potential of metabolically characterized aqueous extract of *Carica papaya* leaves. Pharm. Biol..

[50] Erlund I. (2004). Review of the flavonoids quercetin, hesperetin, and naringenin. Dietary sources, bioactivities, bioavailability, and epidemiology. Nutr. Res..

[51] Li Y., Yao J., Han C., Yang J., Tabassum Chaudhry M., Wang S., Liu H., Yin Y. (2016). Quercetin, inflammation and immunity. Nutrients.

[52] Parthiban P., Siddha M.D., Clinic K.S.M.S., Road K.M. (2016). Analysis of phytochemical constituents and Antimicrobial activity of *Carica papaya*. Int. J. Adv. Res. Biol. Sci..

[53] Kuete V., Simo I.K., Ngameni B., Bigoga J.D., Watchueng J., Nzesse Kapguep R., Etoa F.X., Ngadjui Tchaleu B., Penlap Beng V. (2007). Antimicrobial activity of the methanolic extract, fractions and four flavonoids from the twigs of Dorstenia angusticornis Engl. (Moraceae). J. Ethnopharmacol..

[54] Li Z.Y., Wang Y., Shen W.T., Zhou P. (2012). Content determination of benzyl glucosinolate and anti-cancer activity of its hydrolysis product in *Carica papaya* L.. Asian Pac. J. Trop. Med..

[55] Otsuki N., Dang N.H., Kumagai E., Kondo A., Iwata S., Morimoto C. (2010). Aqueous extract of *Carica papaya* leaves exhibits anti-tumor activity and immunomodulatory effects. J. Ethnopharmacol..

[56] Zhang Z.-S., Wang X.-M., Han Z.-P., Zhao M.-X., Yin L. (2012). Purification, antioxidant and moisture-preserving activities of polysaccharides from papaya. Carbohydr. Polym..

[57] Singh O., Ali M. (2011). Phytochemical and antifungal profiles of the seeds of *Carica papaya* L.. Indian J. Pharm. Sci..

[58] Julianti T., Oufir M., Hamburger M. (2014). Quantification of the antiplasmodial alkaloid carpaine in papaya (*Carica papaya*) leaves. Planta Med..

[59] Kovendan K., Murugan K., Panneerselvam C., Aarthia N., Mahesh Kumar P., Subramaniama J., Amerasan D., Kalimuthu K., Vincent S. (2012). Antimalarial activity of *Carica papaya* (Family: Caricaceae) leaf extract against Plasmodium falciparum. Asian Pac. J. Trop. Dis..

[60] Wabo Poné J., Ngankam Ntemah J.D., Bilong Bilong C.F., Mbida M. (2011). A comparative study of the ovicidal and larvicidal activities of aqueous and ethanolic extracts of pawpaw seeds *Carica papaya* (Caricaceae) on Heligmosomoides bakeri. Asian Pac. J. Trop. Med..

[61] Melariri P., Campbell W., Etusim P., Smith P. (2011). Antiplasmodial properties and bioassay-guided fractionation of ethyl acetate extracts from *Carica papaya* leaves. J. Parasitol. Res..

[62] Ayanfemi A.A., Bukola A.O. (2015). Antibacterial Activity of *Carica Papaya* Leaves and Seeds Extracts on Some Bacteria and their Phytochemical Characterization. Int. J. Bot. Res..

[63] Akujobi C.N., Ofodeme C.N., Enweani C.A. (2010). Determination of antibacterial activity of *Carica papaya* (pawpaw) extracts. Niger. J. Clin. Pract..

[64] Vieira R.H.S.D.F., Rodrigues D.D.P., Gonçalves F.A., De Menezes F.G.R., Aragão J.S., Sousa O.V. (2001). Microbicidal effect of medicinal plant extracts (*Psidium guajava* Linn. and *Carica papaya* Linn.) upon bacteria isolated from fish muscle and known to induce diarrhea in children. Rev. Inst. Med. Trop. Sao Paulo.

[65] Emeruwa A.C. (1982). Antibacterial substance from *Carica papaya* fruit extract. J. Nat. Prod..

[66] Joseph B., Sankarganesh P., Ichiyama K., Yamamoto N. (2014). In vitro study on cytotoxic effect and anti-DENV2 activity of *Carica papaya* L. leaf. Front. Life Sci..

[67] Ahmad N., Fazal H., Ayaz M., Abbasi B.H., Mohammad I., Fazal L. (2011). Dengue fever treatment with *Carica papaya* leaves extracts. Asian Pac. J. Trop. Biomed..

[68] Amazu L., Azikiwe C., Njoku C., Osuala F.N., Nwosu P.J.C., Ajugwo A.O., Enye J.C. (2010). Antiinflammatory activity of the methanolic extract of the seeds of *Carica papaya* in experimental animals. Asian Pac. J. Trop. Med..

[69] Owoyele B.V., Adebukola O.M., Funmilayo A.A., Soladoye A.O. (2008). Anti-inflammatory activities of ethanolic extract of *Carica papaya* leaves. Inflammopharmacology.

[70] Vlachojannis C., Chrubasik-Hausmann S., Hellwig E., Al-Ahmad A. (2015). A Preliminary Investigation on the Antimicrobial Activity of Listerine, Its Components, and of Mixtures Thereof. Phytother. Res..

